# Muscle Cramping During Exercise: Causes, Solutions, and Questions Remaining

**DOI:** 10.1007/s40279-019-01162-1

**Published:** 2019-11-06

**Authors:** Ronald J. Maughan, Susan M. Shirreffs

**Affiliations:** School of Medicine, St Andrews University, St Andrews, Scotland UK

## Abstract

Muscle cramp is a temporary but intense and painful involuntary contraction of skeletal muscle that can occur in many different situations. The causes of, and cures for, the cramps that occur during or soon after exercise remain uncertain, although there is evidence that some cases may be associated with disturbances of water and salt balance, while others appear to involve sustained abnormal spinal reflex activity secondary to fatigue of the affected muscles. Evidence in favour of a role for dyshydration comes largely from medical records obtained in large industrial settings, although it is supported by one large-scale intervention trial and by field trials involving small numbers of athletes. Cramp is notoriously unpredictable, making laboratory studies difficult, but experimental models involving electrical stimulation or intense voluntary contractions of small muscles held in a shortened position can induce cramp in many, although not all, individuals. These studies show that dehydration has no effect on the stimulation frequency required to initiate cramping and confirm a role for spinal pathways, but their relevance to the spontaneous cramps that occur during exercise is questionable. There is a long history of folk remedies for treatment or prevention of cramps; some may reduce the likelihood of some forms of cramping and reduce its intensity and duration, but none are consistently effective. It seems likely that there are different types of cramp that are initiated by different mechanisms; if this is the case, the search for a single strategy for prevention or treatment is unlikely to succeed.

## Key Points

**Table:** 

Exercise-associated muscle cramp (EAMC) is a temporary but intense and painful involuntary contraction of skeletal muscle occurring during or soon after a period of physical activity.
EAMC is highly unpredictable and it seems likely that different mechanisms may operate in different scenarios.
Proposed mechanisms include disturbances of water and electrolyte balance, and abnormal spinal reflex activity.
No prevention strategy or treatment is consistently effective.

## Introduction

Few athletes escape the painful experience of muscle cramps at some stage during their sporting career. Cramps that occur during or soon after a bout of physical activity have been termed exercise-associated muscle cramps (EAMC), and these are commonly experienced as a “painful, spasmodic contraction of the skeletal muscle that occurs during or immediately after muscular exercise” [1].

This review is based in part on a review of the literature using the Web of Science database and the key words ‘cramp’, ‘muscle’ and ‘exercise’. Titles and abstracts of the 379 results returned were screened for relevance. The same search on PubMed returned 236 items. However, although the timespan for the Web of Science was set to 1900–2019, this search revealed no publications prior to 1966. No date was specified for the PubMed search, but the earliest relevant publication returned by the search appeared in 1960. These searches thus excluded all of the older literature, and this perhaps explains why most publications continue to ignore this. Earlier publications were identified from various sources.

Several surveys have attempted to identify the prevalence of EAMC in different sports populations, but comparing results across studies is hampered by different definitions and different measurement periods, and also by the use of different assessment tools. Nonetheless, EAMC has been reported to affect 67% of triathletes during or after training or racing [2], 18–70% of marathoners or endurance cyclists [3–5], and 30–53% of American football players [6, 7]. Although seemingly suggesting that cramp is common, these data are a mixture of incidence rates in single events and lifetime incidence. Most often, cramping is a relatively minor inconvenience: Schwabe et al. reported the incidence of serious muscle cramping to be less than one per thousand runners in a large cohort (65,865 runners) of participants in half-marathon and ultra-marathon events [8]. To put these data in perspective, Abdulla et al. reported that among an outpatient sample aged 65 years or older, 50% of outpatients experienced frequent muscle cramps [9], and that another survey of a similar population reported a similar prevalence of 56%, with half having cramps occurring at least once per week [10]. About 7–12% of patients with amyotrophic lateral sclerosis (ALS), a progressive, fatal neurodegenerative disorder, present with muscle cramping [11].

The statistics on EAMC from athlete populations do not reveal the fact that for some of those afflicted, it may be a rare occurrence—perhaps only one or two incidents over the course of a whole career, and therefore mostly of negligible impact—while others may be affected much more frequently and much more severely. The intensity and duration of cramps can vary greatly, from a minor spasm that resolves spontaneously within a few seconds, to the whole-body ‘lock up’ lasting several minutes that some athletes describe. In severe cases, the muscle pain may persist for hours or even days after the acute contraction has resolved, and may result in an inability to train or compete. At worst, repeated episodes can result in a premature end to an athlete’s career.

There are many different potential causes of muscle cramps, most of which are not associated with exercise but with a range of clinical conditions or the use of drugs for the treatment of those conditions [12–14]. Even within the narrow area of EAMC, the highly localised cramp in the calf that afflicts the football (soccer) player late in the game is very different from the whole-body cramps that some American football players and tennis players describe and that have been reported in some industrial settings. These in turn are different from the cramp that afflicts small muscles used in repetitive exercise, such as the hand in writers or typists [15]. Cramps typically occur spontaneously and may or may not occur predictably. Some cramps are associated with fasciculations or other prodromal symptoms, but there may be no warning in other cases [12]. Cramp in some small muscles can be induced in the laboratory, but not all cramps can be induced reliably and not all individuals are susceptible, making them difficult to study. Likewise, some cramps occur early on during exercise, while some occur only after prolonged periods of exercise; others still occur some minutes or even many hours after exercise. It is not clear that the mechanisms underpinning these different types of cramp are the same.

This uncertainty is reflected in the conclusion of several recent reviews that the causes of EAMC, and therefore the treatment options, remain uncertain [16, 17]. Two main hypotheses have been proposed and continue to be debated: a disturbance of water and salt balance, and a neurological cause resulting in sustained abnormal discharge of motor drive to the afflicted muscles [18]. Each of these has some support, but neither can fully explain the nature of EAMC.

## Risk Factors for Exercise-Associated Muscle Cramp (EAMC)

Although EAMC has been observed in both training and competition in almost every type of sport, it does seem from the surveys cited above to be more associated with endurance-type activities and in team sports. An analysis of the evidence of cramp among American football players showed that the great majority (95%) occurred during periods of hot weather: EAMC occurred most often during the first 3 weeks of practice, when fitness and acclimation levels are likely to be lowest and when the training load is often the highest [19]. The incidence of heat cramps was 37% during the first week of the training camp, then 27%, 18% and 4% in the succeeding weeks. Notwithstanding these observations, the incidence of EAMC may also be high in endurance events taking place in cool or cold environments: Maughan found that 15 of 92 (18%) runners experienced cramp during a single marathon race taking place at 10–12 °C [3]. Most cases occurred in the later stages of the race, after an average of 35 km had been completed: no cases were reported to have occurred before 24 km, and 5 of the 15 instances occurred during the last 1.5 km.

Schwellnus and colleagues have made a number of attempts to characterise the primary risk factors predisposing to cramp in endurance events. Furthermore, Schwellnus suggested that EAMC in marathon runners is associated with high intensity, long duration, and hilly terrain, which can lead to ‘premature muscle fatigue’ in competitors with a history of cramping [20]. It is not immediately obvious what is meant by ’premature’ fatigue and how this might differ from the fatigue that is an inevitable consequence of participation in such events [20]. Schwellnus et al. reported that, in a prospective cohort study in 210 Ironman triathletes, independent risk factors for EAMC were a history of the condition and competing at a higher than usual exercise intensity, but that dehydration and serum sodium changes did not predict EAMC [21]. Manjra et al. analysed data from 1300 marathon runners and found that risk factors included those common to all participants in marathon races, including long distance (> 30 km) and the presence of fatigue, but also running at a faster pace than was normal in training [5]. Other risk factors included older age, a longer history of running, higher body mass index (BMI), shorter daily stretching time, irregular stretching habits, and a positive family history of cramping.

In a more recent analysis of cross-sectional data from almost 16,000 participants in two races over a distance of 21.1 km and 56 km, Schwellnus et al. identified a number of differences between runners who reported a history of EAMC (*n* = approximately 3000) and a control group with no such history (*n* = approximately 13,000) [22]. Factors associated with a history of EAMC included underlying chronic disease (including cardiovascular, respiratory, gastrointestinal, nervous system, kidney, bladder and haematological disease), as well as cancer, allergies, regular medication use, and a history of injury. More experienced runners were also at greater risk. Whether some underlying common factors underpin these associations is not at present clear.

In what seems to be the largest survey to date, but published only as an abstract, Swanevelder et al. analysed data from an online pre-race medical screening questionnaire completed by 41,698 distance runners who completed either a 21.1 km or 56 km run [23]. The investigators considered independent risk factors associated with EAMC (model 1: binary outcome), and risk factors associated with severe EAMC (model 2: defined as recurrent cramping history), and found some rather inconsistent outcomes. For model 1 (binary outcome), significant risk factors for EAMC were males, age > 40 years, increased BMI, history of any disease of the gastrointestinal tract or kidney/bladder, chronic or regular medication use, history of a running injury in the last 12 months, running the 56 km race, recreational running for < 5 years, training/racing < 3 times/week, and slower runners (> 6 min/km). In model 2 (recurrent cramping history), the authors said that “EAMC was associated with all the risk factors for EAMC in model 1, but also included a history of any cardiovascular disease (CVD) symptoms. In model 2, a lower BMI and running in 21.1 km race were also specific risk factors for severe EAMC. Training volume and pace weren’t risk factors in model 2”. There seem to be some mutually contradictory statements here.

## Possible Causes of EAMC

Two main causes for muscle cramps have been proposed and, depending on which an individual subscribes to, the choice of prevention and treatment strategies will be determined. This suggests an either/or dichotomy, and this is how the literature is often presented, with loud voices expressing strongly held views on either side [24, 25]. It should be recognised though that the picture is not at all clear, and the evidence on both sides of the debate is weak. It is unlikely that a single mechanism can account for all cramps in all situations, therefore the search for a single causal mechanism is probably futile. It follows from this that strategies for the prevention and treatment of the condition are also unlikely to be one-dimensional. However, whatever the primary cause, it is clear that cramp is accompanied by active contraction of the afflicted muscle, as evidenced by high levels of muscle electrical activity [26].

### Disturbances of Hydration and Electrolyte Balance

The role of changes in hydration status and electrolyte balance as a factor in the aetiology of EAMC was dismissed by Schwellnus, who said that “Scientific evidence in support of the ‘‘electrolyte depletion’’ and ‘‘dehydration’’ hypotheses for the aetiology of EAMC comes mainly from anecdotal clinical observations, case series totalling 18 cases, and one small (*n *=10) case–control study” [25]. This assessment of the evidence has been repeated in many subsequent publications: for example, Qiu and Kang wrote that “its [i.e. the electrolyte imbalance-and-dehydration theory] supporting evidence comes mainly from anecdotal observations and case reports” [27]. There may however be more evidence than these authors admit.

The strongest evidence that sweat-related electrolyte imbalances are a factor in some muscle cramps is found in the large-scale observational and prospective studies of industrial workers—mainly studies on miners, ship’s stokers, construction workers and steel mill workers that were conducted in the 1920s and 1930s—where administration of saline drinks or salt tablets was able to greatly reduce the incidence of cramps [28–32]. These studies were inevitably limited by the methods available at the time, but they did have the advantage of access to large populations and the keeping of careful medical records related to productivity. It is easy to dismiss much of the older literature, but some of the observations were extensive and meticulous. They should also be read in the context of the normal publishing conventions of the time.

Although methodologies were limited, some of the observations were acute and sometimes remarkably prescient. For example, Moss published an extensive report in which he documented cases of cramp among coal miners and the factors that may have contributed to the development of these cramps [28]. He attributed the onset of cramps, which in some cases were seriously debilitating, to (1) high air temperatures; (2) excessive drinking of water caused by dryness of the mouth and throat; and (3) continued hard work.

He also observed that cramps tended to occur during the second half of a working shift and in men who were less physically fit, thus implicating not only sweat losses but also fatigue in the aetiology. It should be noted that cramp was not attributed to dehydration or increased serum electrolyte concentrations, but rather “to a form of water poisoning of the muscles brought about by the combination of great loss of chloride by sweating, excessive drinking of water, and temporary paralysis of renal excretion” [33]. Chloride was normally measured in body fluids as there was no good assay for sodium at the time, but there is a close relationship between sodium and chloride concentration in sweat [34]. This does not implicate dehydration, as most of the later writers say (e.g. Bergeron [24]), but rather inappropriate, and perhaps excessive, intake of plain water in combination with large losses of electrolytes in sweat. Schwellnus refers to ‘dehydration’ and ‘electrolyte depletion’ theories [25], while Qiu and Kang say that “this theory suggests that overly sweating and thus loss of electrolytes can cause muscles and nerves that innervate them to malfunction, thereby producing muscle cramps” [27]. This is not a true reflection of the theories proposed during the 1920s and 1930s.

It is also not correct to say that there have been no large-scale prospective studies to assess the role of water and salt balance in the aetiology of muscle cramps. Dill et al. reported the findings of intervention studies carried out at the site of construction of the Hoover Dam and in the steel mills of Youngstown, Ohio [32]. At both of these locations, large numbers of men undertook hard physical work in extremely hot environments on a daily basis. They found that those suffering from cramp displayed the following characteristics: (1) dehydration; (2) lowered concentration of sodium and chloride in blood plasma; (3) little or no sodium or chloride in urine; (4) increased serum protein concentration; (5) increased red cell count; and (6) normal osmotic pressure.

This presents a complex picture: some of these findings are typical of dehydration (1, 4 and 5), while others are consistent with overhydration (2, 3). However, they also reported that injection of isotonic saline normalised the blood profile and brought immediate relief from the symptoms. In the largest intervention study, reported in the same paper, they added saline to the water given to the 12,000 men employed in one of the mills, while those at neighbouring mills continued to be provided with plain water; this was effective in almost completely abolishing cases of muscle cramp, although in previous years, and at other mills in the same year where plain water was given, up to 12 cases of cramp required hospitalisation in a single day.

In a controlled environment, severe restriction of dietary sodium intake can result in hyponatraemia and may be associated with generalised skeletal muscle cramping in the absence of exercise [35]. Some more recent studies have assessed changes in hydration status and plasma electrolyte concentrations in athletes who have experienced muscle cramps; these studies have included marathon runners [3], participants in a 56 km road race [36], competitors in an Ironman triathlon [37], and participants in a 161 km ultramarathon [38]. None of these showed any association between cramp and serum electrolyte changes, but it is important to note that serum electrolyte concentrations may be of little relevance. Local intracellular and extracellular electrolyte concentrations may be relevant as they will affect the resting membrane potential of both muscle and nerve, but it is unlikely that changes in plasma concentrations can track these changes; there is good evidence that changes in the plasma concentration of these electrolytes do not reflect local intramuscular changes during either intense or prolonged exercise [39, 40]. It is also the case that blood samples have usually not been collected at the time of cramping, but only later, usually once the cramping has resolved; in some cases, this was several hours after resolution of the cramps, therefore the absence of any association is perhaps not surprising. Schwellnus et al. acknowledged that disturbances in electrolyte concentrations can lead to alterations in neuromuscular excitability, and this may have a role in the generalised skeletal muscle cramping reported in some industrial contexts, but argue that most EAMC affects only the muscles involved in the exercise task, suggesting that systemic disturbances must interact with local changes occurring within the active muscles [1].

There is some experimental evidence that individual athletes who lose large amounts of salt in their sweat may be more prone to muscle cramps. Unlike the earlier large-scale industrial records, this evidence does derive primarily from small studies, case reports and anecdotal reports, and is therefore inevitably rather weak [41, 42]. Stofan et al. found that sweat sodium losses during training sessions were larger in cramp-prone football players (*n* = 5) than in a group of players with no history of EAMC [41]. Subsequently, the same research group investigated a reference group of American football players (*n* = 8) without a cramping history, and a cramp-prone group (*n* = 6) [42]. Whole blood sodium concentration (as stated by the authors, but in reality this is plasma sodium concentration) remained unchanged after training in the control group (138.9 ± 1.8 to 139.0 ± 2.0 mmol/L), while it tended to decline (137.8 ± 2.3 to 135.7 ± 4.9 mmol/L) in the cramp-prone players. Additionally, three subjects in this group recorded values below 135 mmol/L. Those in the cramp-prone group consumed a greater percentage of their total fluid as plain water rather than electrolyte-containing sports drinks (although the difference in sodium intake was small) and had a higher sweat sodium concentration (52.6 ± 29.2 mmol/L vs. 38.3 ± 18.3 mmol/L), thus incurring a greater sodium deficit over the course of the training session.

In support of a role for disturbances of water and salt balance as a causal factor, Ohno and Nosaka showed that a body fluid deficit of 3% of body mass induced by intermittent sauna exposure without exercise increased the number of subjects who developed EAMC during a muscle cramp test in the toe flexors, but not in the knee extensors [43]. Jung et al. had participants perform a fatiguing protocol in the calf muscles to induce EAMCs. In one trial, subjects consumed a carbohydrate electrolyte drink at a rate similar to sweat rate, while in the other trial, no fluid was consumed and mild (1% loss of body mass) hypohydration developed [44]. Nine participants experienced cramps in the carbohydrate–electrolyte trial, compared with seven in the hypohydration trial. Of the seven individuals who had EAMC in both trials, time to onset was more than doubled in the carbohydrate–electrolyte trial (36.8 ± 17.3 min) compared with the hypohydration trial (14.6 ± 5.0 min). Subjects who experienced cramps sweated more (2.0 ± 0.9 L/min) than those who did not (1.3 ± 0.6 L/min). It is not clear whether there was any treatment order effect in these studies that might have confounded the results (this is discussed further below).

Although numerous papers have disputed the findings above, two recent publications seem likely to reopen the debate on the role of disturbances of water and salt balance in the development of muscle cramps. Ohno et al. systematically investigated the susceptibility of voluntarily-induced EAMC in hamstrings after hypohydration of 1, 2 and 3% of body mass induced by sauna exposure without exercise [45]. No EAMC occurred in the nine participants in the control condition or after 1% dehydration; three subjects experienced EAMC in the 2% and six in the 3% condition. In the study by Lau et al., 10 men ran downhill in a hot environment until they lost 2% of their initial body mass [46]. Ten minutes after completing the run, they ingested either plain water or a commercially available oral rehydration solution (ORS) containing sodium (50 mEq/L), chloride (50 mEq/L), potassium (20 mEq/L), magnesium sulphate (2 mEq/L), lactate (31 mEq/L) and glucose (18 g/L) in a volume equal to the mass lost. Susceptibility of the calf muscles to electrically-induced cramp was assessed by a threshold frequency (TF) test applied at baseline before running, immediately after running, and 50 and 80 min after drink ingestion. Muscle cramp susceptibility assessed by TF did not change from baseline to immediately after running in either condition, but TF decreased after water intake by 4.3 Hz (at 30 min) and 5.1 Hz (at 60 min post-run), but increased after ORS intake by 3.7 and 5.4 Hz, respectively. The investigators reported that serum sodium and chloride concentrations decreased after water intake but were maintained after ingestion of the electrolyte-containing drink.

In accord with the mechanisms proposed by Moss, Haldane and others in the 1920s, these results suggest that the combination of sweat loss and water intake makes muscles more susceptible to electrical simulation-induced muscle cramp, but the susceptibility to muscle cramp decreases when a drink with a high electrolyte content is ingested. It is interesting to note that cramping is a recognised accompaniment of hyponatraemia (defined as a serum sodium concentration < 135 mmol/L) in clinical settings [47]. However, the extensive literature on exercise-associated hyponatraemia generally makes no mention of muscle cramping [48].

While cramp is often associated with large sweat losses during prolonged exercise in the heat, it also occurs in cool environments with little or no sweat loss, suggesting that sweat loss alone and the consequent disturbances of electrolyte balance cannot account for all cramps. Notwithstanding these observations, there is overwhelming evidence from large-scale industrial settings that cramping occurs more frequently in environments that are hot (although not necessarily humid) and where sweat losses are high [28, 31]. Supporting evidence that disturbances of electrolyte balance may be implicated in muscle cramps can be found in some non-exercise contexts. For example, the use of low-sodium dialysis fluids during maintenance dialysis may provoke cramping in renal patients [49], and normalisation of plasma osmolality and sodium concentration by use of the sodium profiling technique can significantly reduce the frequency of cramping during dialysis [50]. Whether this is relevant to the exercise situation though is uncertain.

### Altered Neuromuscular Control

The idea that the cause of cramp is neurological rather than being related directly to events occurring within the muscle is not a new one. Telegraphists’ cramp, affecting the small muscles of the hand involved in the repetitive movements of those operating a Morse code instrument, was the subject of a UK Parliamentary enquiry that published its findings in 1911 [51]. The Committee wrote that “Some authorities have regarded it [i.e. telegraphists’ cramp] as a muscular disorder; others as a disease of the peripheral nervous system; others as a disease of the central nervous system”. They further wrote that “After careful consideration of these antagonistic theories of telegraphists’ cramp, and examination of a number of telegraphists affected with the disease, the Committee accept the last-named view; namely, that telegraphists’ cramp is a disease of the central nervous system, and is the result of a weakening or breakdown of the cerebral controlling mechanism in consequence of strain upon a given set of muscles”. As will be seen below, this is remarkably similar to the proposed mechanism in experimentally-induced muscle cramps. However, the findings of the Parliamentary enquiry seem to have been largely forgotten, along with much of the older literature.

**Fig. 1:**
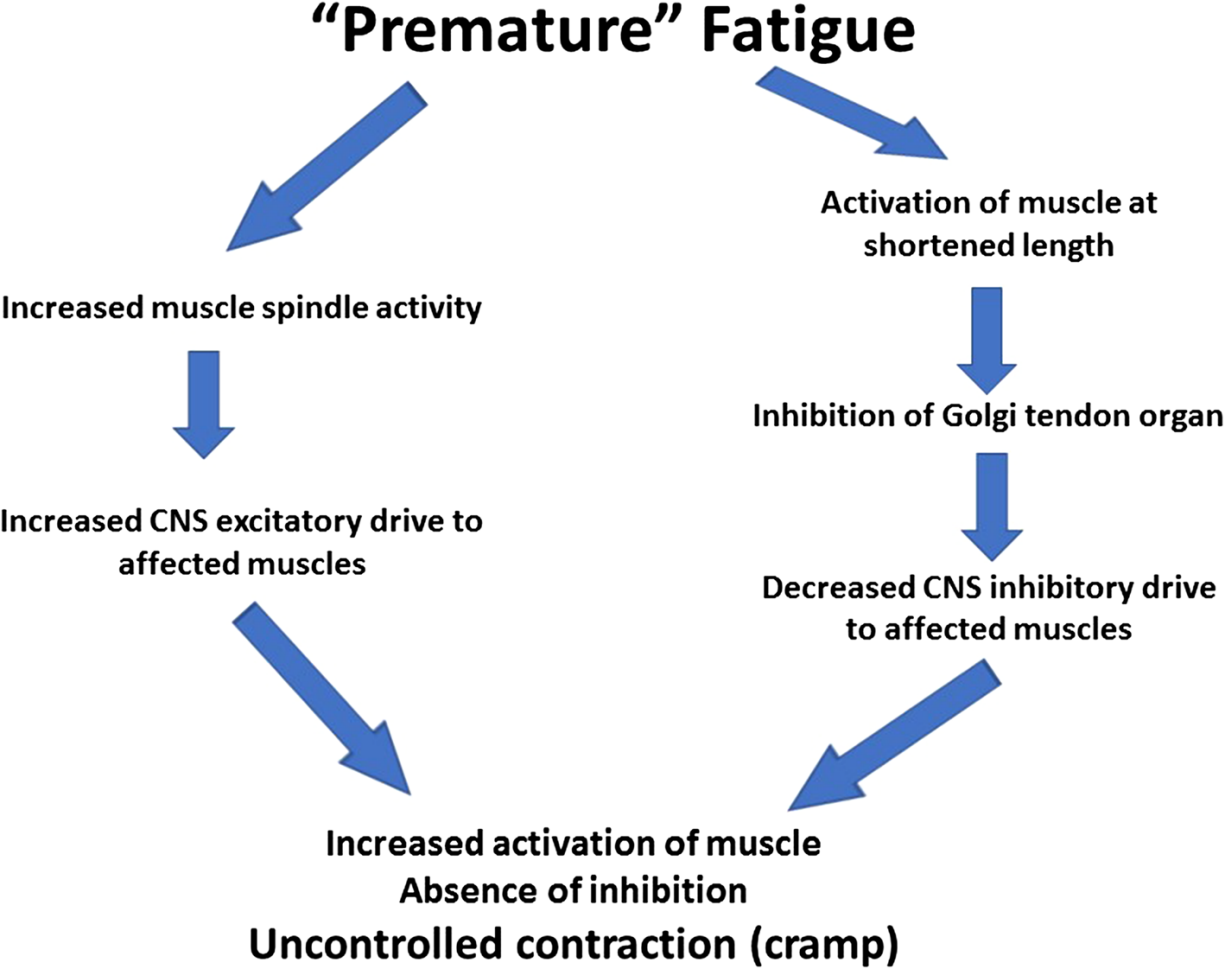
Postulated abnormal spinal control of motor neuron function during exercise-associated muscle cramp. Based on a proposal by Schwellnus et al. [1]. *CNS* central nervous system

As evidence accumulated in the 1980s and 1990s that cramp often occurred during exercise in the absence of substantial sweat losses or of gross disturbances in electrolyte balance, an alternative causation was sought. Schwellnus et al. hypothesised that cramp is caused by “sustained abnormal spinal reflex activity which appears to be secondary to muscle fatigue” [1]. In particular, EAMC was ascribed to an abnormality of sustained alpha motor neuron activity due to an abnormality of alpha motor neuron control at the spinal level, but this still does not identify the cause of this abnormality. Muscle fatigue was implicated through an excitatory effect on the muscle spindle afferent activity (type Ia and II) and an inhibitory effect on the type Ib Golgi tendon organ afferent activity (Fig. 1). Circumstantial evidence in support of this suggestion arose from the observation that passive stretching of the muscle during an episode of cramp may alleviate the symptoms as a result of an autogenic inhibition by the tendon organ reflex [52]. However, this still does not explain why cramp is not an inevitable consequence of exercise that causes fatigue, why it appears to occur more frequently in environments that impose high thermal stress, or why some individuals are affected while others are not.

The strongest evidence for an altered neuromuscular control is from laboratory studies of small muscles in humans and in animal models. In each of these two different scenarios, a story can be made, but in each case the story is incomplete. Because EAMC is notoriously unpredictable, laboratory models have been developed where cramp can be induced more reliably, whether by voluntary activation of muscles or by electrically-evoked contractions. It has been reported that cramping occurs more frequently when the muscle is activated while it is already shortened [1] (although no evidence to support this statement was presented). Various forms of this experimental model have been used in laboratory studies of cramping, even though this may not reflect the movement patterns of athletes. This is consistent with the proposal of Schwellnus et al. [1] as outlined above, as a reduction in the tension in the muscle tendon will reduce the inhibitory feedback from the Golgi tendon organ; this in turn has the potential to increase the motor drive to the alpha motor neuron. Consistent with this proposal, Khan and Burne [26] found that cramp induced by voluntary maximal activation of the gastrocnemius while it was held in a shortened position could be inhibited by electrical stimulation of tendon afferents in the cramped muscle. However, even under conditions that favoured cramping, 5 of their 13 subjects could not induce cramping, and in a further two it did not persist long enough for measurements to be made.

Athletes who are prone to muscle cramps are reported to demonstrate a lower threshold for muscle cramps evoked by electrical stimulation of motor nerves [53, 54]. Blocking of the motor nerves with anaesthetic does not abolish these electrically evoked cramps, but when the nerve is blocked, a greater stimulation frequency is required to induce cramping and cramp duration is reduced; altered motor unit discharge characteristics are consistent with the existence of a positive feedback loop involving afferent input from affected muscles and motor drive to those muscles [55].

Strong objections to the dehydration/electrolyte loss theory have been raised by studies that have provided fluids to prevent dehydration and found that this does not affect the onset of electrically-evoked cramps [56, 57]. However, these findings are contradicted by other studies referred to above [43–46]. It should be noted that marked hypernatraemia developed as a result of dehydration in the studies of Miller et al. [56] and Braulick et al. [57], and this may be protective against the development of cramp [46]. Fatigue alone is also unlikely to be the cause, although it may be a contributing factor. In marathon runners, cramp tends to occur more frequently towards the end of races [3, 5]; however, everyone is fatigued in the later stages of endurance events such as a marathon race, but relatively few experience muscle cramps. The nature of the fatigue that occurs in sprinters is very different from that experienced towards the end of a marathon race, but cramp may occur in either situation.

Therefore, rather than focusing on an either/or approach, there are good reasons to suggest that different mechanisms may apply in different situations. We are all inevitably influenced by our own experiences and these may bias us towards one cause as being more likely or more common than another, but the key issue is how to treat or prevent an attack. With regard to treatment and prevention, it is important to note that a plausible mechanism can help to identify effective treatments, but it is not necessary to understand mechanisms to know if a treatment is effective or not.

## Possible Preventive and Treatment Strategies

The early studies of muscle cramping that occurred in industrial settings identified large sweat losses and ingestion of large volumes of plain water as factors contributing to muscle cramping, therefore it is not surprising that ingestion of salt was proposed as a prevention strategy [33, 58]. The strongest evidence for the efficacy of this strategy is found in the work of Dill and colleagues [32], where large-scale prospective studies showed that the addition of salt to drinking water was effective in reducing the rate of cramping.

Schwellnus et al. said that “The treatment of acute muscle cramps is passive stretching” [1]. In support of this, they showed data from a single runner in whom stretching resulted in a dramatic decrease in the electromyographic activity of the affected muscle. The same group also suggested that ‘irregular stretching habits’ were associated with an increased risk of cramping [5]. They later hypothesised that variants in genes that code for connective tissue components of muscle may influence the susceptibility to EAMC [59]. To test this hypothesis, they recruited 116 ultraendurance athletes with a recent self-reported history of EAMC, and 150 participants who had never experienced EAMC. As per the hypothesis, the COL5A1 CC genotype was significantly overrepresented (*p* = 0.031) among the control group (21.8%) compared with the EAMC group (11.1%). However, none of the other related genes showed a differential distribution. A review by Nelson and Churilla stated that there is ‘strong evidence’ that passive stretching is the most effective treatment for muscle cramping, but did not identify this evidence [60]. More recently, Panza et al. tested the possible association between acute static stretching of the muscle and prevention of cramping, using an experimental model whereby cramp was induced in the flexor hallucis brevis muscle by electrical stimulation with the muscle held in a shortened position [61]. In a crossover study, static stretch was compared with a no-stretch condition; the cramp TF increased in both the control and stretching conditions, with no difference between conditions. Miller et al. subsequently reported similar findings [62].

There is a long history of the use of folk remedies for the prevention and treatment of muscle cramps, and many of these have included compounds that have a strong or bitter taste, including pickle juice, mustard, quinine, vinegar and various spices and herbs. Even homeopathic cures are reported to be effective, with anecdotal support from athletes often being used to promote these products, suggesting that both the placebo effect and athlete belief may play a powerful role [63]. As with other interventions, these have proved difficult to evaluate as muscle cramps generally resolve spontaneously before any intervention can be implemented. However, in the human model of electrically-invoked cramp, pickle juice (which has a high salt content and a sharp taste imparted by the acetic acid content) was reported to be effective in reducing the duration of cramps. Miller et al. found that cramp duration was reduced by about 37% on average when 1 mL of pickle juice was ingested 2 s after induction of cramping, compared with a trial where water was ingested (85 ± 19 s vs. 134 ± 16 s, respectively; *p* < 0.05); the intensity of cramping was not affected [64]. The same authors had previously shown that ingestion of small volumes of pickle juice had no measurable effect on plasma concentrations of sodium, potassium, magnesium or calcium concentration, or on plasma osmolality and plasma volume [65]. The authors proposed that, in the absence of any effect of the ingested pickle juice on circulating electrolyte concentrations, the mechanism by which pickle juice shortened cramp duration involved activation of receptors in the oropharyngeal region that resulted in a reduced firing rate of alpha motor neurons that innervate the affected muscle. However, it is important to note that this was not a study of EAMC, but of cramping induced by electrical stimulation during maximal voluntary contraction of a small muscle in the sole of the foot that was held in a shortened position; this cannot be taken as evidence of efficacy in the treatment of EAMC. However, this and the results of other similar studies, raise some interesting questions; crossover designs involve using the same subjects in treatment and placebo trials, usually in the case of a single treatment, with half receiving treatment before placebo and the order reversed in the other half. The statistical analysis applied in the study by Miller et al. [64] assumes that there was no treatment order effect, but we cannot be sure that this is true, with only 1 week for recovery between experimental trials [66]. The authors of this and other studies involving similar experimental designs should have reported whether the cramp intensity and cramp duration were different between the first and second exposures, and should perhaps also have habituated the subjects to the electrical stimulation process prior to the experimental trials. The importance of this is highlighted by a recent publication showing that repeated exposures to electrically-evoked cramps induce a long-lasting increase of the cramp TF in healthy subjects [67]. These authors induced EAMC in the gastrocnemius medialis of one leg twice a week, while the opposite leg served as the control leg; after four cramp training sessions, the cramp threshold frequency (CTF) increased in the intervention leg but not in the control leg. This same consideration of course applies to many other laboratory studies of electrically-evoked cramping, but becomes particularly acute when, as in the study of Miller et al. [64], a large difference between conditions occurs in the first trial, with possible consequences for the succeeding trial.

Quinine has been considered to have a possible role in the prevention of cramps. There is little research specific to EAMC, but a 2015 Cochrane review concluded that ingestion of quinine (200–500 mg daily) reduces cramp number and cramp days (low-quality evidence) and reduces cramp intensity (moderate-quality evidence), but has no effect on cramp duration [68]. They reported some evidence that ingestion of theophylline in combination with quinine improved cramps more than quinine alone. They also drew attention to the risks of adverse events associated with quinine use. These conclusions are in general agreement with those of an earlier review [13].

A recently launched product has claimed that cramp can be prevented or treated by activation of transient receptor potential (TRP) in the mouth [69], although this has not been supported by other research [70]. TRP receptors form a family of 28 related ion channels that are thought to be important for mediating the sensations of taste and pain. The TRPV1 and TRPA1 channels are stimulated by the active components of spicy foods such as chilli peppers or wasabi. It may be that evidence will emerge to support the product, but there are some questions about the science. There is no doubt that unpleasant (or pleasant) sensations in the mouth will induce electrical activity in some regions of the brain, but there are some gaps in the chain of events between stimulation of oropharyngeal receptors and the inhibition of activity in motor nerves. TRPV1 is activated by capsaicin if the local pH is < 6 [71], but it is by no means certain that such a pH will be reached in the mouth after ingestion of this product. Having said that, it is clear that ingestion of foods containing chilli, ginger and many other foods has powerful effects on receptors in the mouth and elsewhere. Anyone who has put raw chilli in their mouth or near their eye will be aware that this causes not only pain and irritation but also a variety of physiological responses. Whether these signals can disrupt the electrical activity associated with spontaneous muscle cramps remains uncertain.

## Conclusions

Exercise-associated muscle cramp is a relatively common occurrence in a range of sport and exercise activities. Onset is generally unpredictable, and the intensity and duration of muscle spasms are highly variable. Spontaneous muscle cramping in occupational settings involving hard physical effort suggests that high ambient temperature and large sweat losses accompanied by the ingestion of large volumes of plain water may be risk factors, and there is some evidence that the risk is reduced by the addition of salt to ingested fluids. Laboratory models of cramp involve either voluntary or electrically-evoked activation of muscle held in a shortened position. These studies have produced mixed results regarding the effects of disturbances of water and salt balance on the risk of cramping; however they do suggest that, at least in this model, sensory organs in the muscle invoke abnormal reflex activity that results in sustained motor drive to the afflicted muscles. There may be different mechanisms at work in different situations, and there is no conclusive support for any of the proposed mechanisms. Preventive and treatment strategies are not uniformly effective.
